# First Record of Twin and Triplet Embryos Found in the Clutch of a Wild Burmese Python in Southern Florida

**DOI:** 10.1002/ece3.72040

**Published:** 2025-08-25

**Authors:** Génesis Aponte Santiago, Judith E. Baird‐Lujano, Jacquelyn C. Guzy, Derrick G. Biglin, John‐Kaarli M. Rentof, George F. Bancroft, Christina M. Romagosa, Matthew McCollister, Kristen M. Hart

**Affiliations:** ^1^ U.S. Geological Survey, Wetland and Aquatic Research Center Davie Florida USA; ^2^ Cherokee Nation System Solutions, Contracted to the U.S. Geological Survey Davie Florida USA; ^3^ University of Florida and U.S. Geological Survey Intern Program, Stationed in Big Cypress National Preserve Ochopee Florida USA; ^4^ Department of Wildlife Ecology and Conservation University of Florida Gainesville Florida USA; ^5^ National Park Service, Big Cypress National Preserve Ochopee Florida USA

**Keywords:** clutch, egg, embryonic development, invasive species, python, reproduction, snake

## Abstract

Triplet embryos observed from within an egg oviposited by a wild adult female Burmese python in southern Florida. All three embryos were attached to the yolk and found deceased and at differing stages of development.

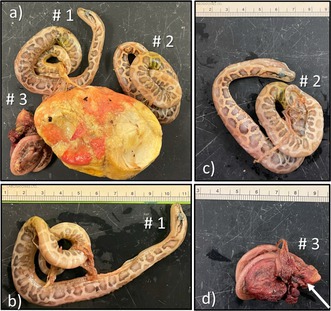

The Burmese python ( *Python molurus bivittatus* ) is an egg‐laying large constrictor native to southeastern Asia; however, there is an established invasive population inhabiting much of southern Florida (reviewed in Guzy et al. 2023). Wild Burmese python morphology and reproductive parameters have been summarized from the necropsy of thousands of specimens, including gravid females (Currylow et al. 2022), and observations made of anomalous pythons that have oviposited but still retained eggs (Anderson et al. 2022). However, thus far, there have been no observations of post‐ovipositional egg abnormalities. In wild lizards, turtles, and crocodiles, there have been reports of twins found in eggs (Eckert 1990; Eckhardt 2018; Vega and Siroski 2021; Rodríguez‐Cabrera et al. 2022; Di Marzio et al. 2023; Kar et al. 2024; Ugemuge et al. 2024; Warner et al. 2024). However, observations from snakes are more limited and are primarily from captive‐bred individuals (e.g., Marion 1980; Clark and Tytle 1983; Manimozhi et al. 2006) or wild‐caught females that oviposited in captivity (Dieckmann et al. 2014; Rodríguez‐Cabrera et al. 2022). In some cases, these twins survived for some time in captivity, including a pair of twins from a Cuban boa ( *Chilabothrus angulifer* ); although if born in the wild, authors suggest they would not have survived given their small size and presumed increased vulnerability to predation (Rodríguez‐Cabrera et al. 2022). As far as we are aware, there are no data on the survival of wild twins in snakes. Embryos of lizard twins rarely appear to hatch or survive long‐term (Di Marzio et al. 2023). The first record of twins from a Burmese python was documented from a captive‐bred clutch that produced two eggs, each with a pair of twins, one of which contained conjoined individuals; neither set of twins survived to hatch (Clark and Tytle 1983). To date, no instances of triplets in snakes have been documented in the literature. Any reports of triplets in reptiles appear limited to lizards (Eckhardt 2018). Here, we document the first instance of a snake, a wild Burmese python, laying eggs from one clutch containing both twin and triplet embryos.

On 17 May 2024, a radio‐tagged female Burmese python (313.7 cm snout‐vent length, SVL) oviposited a clutch of 37 eggs within a hardwood hammock in Big Cypress National Preserve, in Ochopee, Florida (Figure 1, Table 1). Estimates of incubation time for wild Burmese pythons in Florida range from 55 to 63 days; (Currylow et al. 2022, Guzy et al. in review). We monitored this clutch in situ as part of a long‐term assessment of python reproduction and survival. On 14 July 2024, prior to the estimated hatch date, the female was removed from the clutch of eggs to allow for the placement of an enclosure (i.e., polyvinyl chloride frame encased in two nested mesh laundry bags) around the clutch to prevent hatchling escape. During this time, non‐viable eggs were removed, and trail cameras were used to remotely monitor the eggs. On 17 July 2024, eggs began hatching in the enclosure, and hatchlings were collected, weighed, and measured as they emerged over the course of 5 days until 22 July 2024. Of 37 oviposited eggs, 32 developed normally and hatched, three were unfertilized, and two were non‐viable and contained embryos that died before hatching (Figures 2 and 3; Egg A and Egg B; Table 1; Guzy et al. 2025). Egg A contained twin embryos, whereas Egg B contained a set of triplet embryos, and all embryos were deceased and in various stages of development.

**FIGURE 1:**
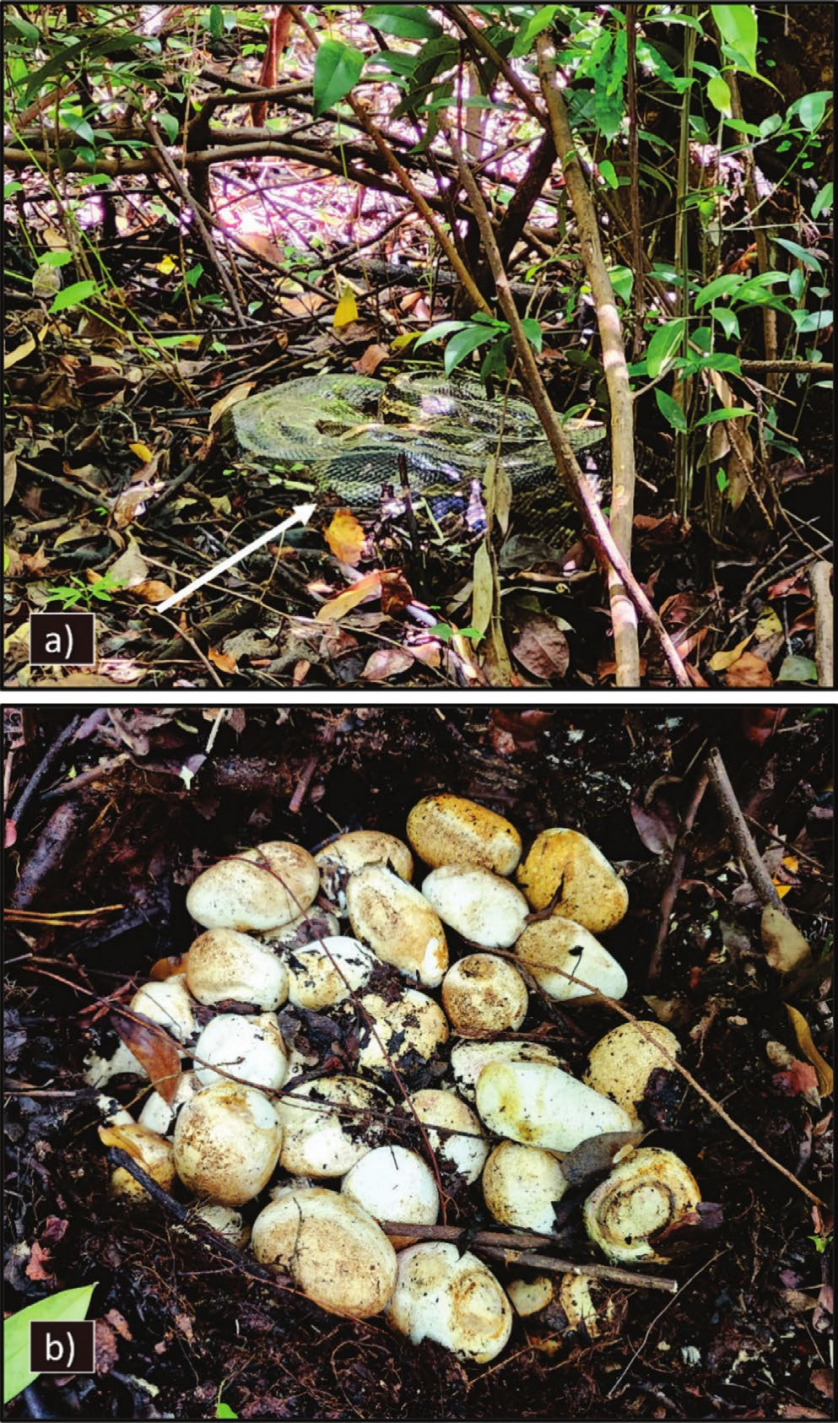
Wild adult female Burmese python ( *Python molurus bivittatus* ) (a) in Big Cypress National Preserve, Collier County, FL, USA, brooding a clutch of 37 eggs (b) oviposited on 17 May 2024.

**TABLE 1:** Mass and length of embryos and hatchlings from a clutch of 34 eggs (32 single embryo, 2 multi‐embryo) oviposited by a wild adult female Burmese python in southern Florida.

		Sample size	Sex	Mass (g)	Snout‐vent length (cm)	Tail length (cm)	Total length (cm)	Yolk mass (g)
Parent python		1	Female	20,600	333.1	39.5	372.6	—
Twin #1		1	Female	45	—	—	—	—
Twin #2		1	Female	48.3	34.3	5.9	40.2	—
	Sum			93.3	—	—	—	33.2
Triplet #1		1	Unknown	14.2	23.2	3.7	26.9	—
Triplet #2		1	Unknown	15.1	24.2	3.9	28.1	—
Triplet #3		1	Unknown	5.6	—	—	—	—
	Sum			34.9	—	—	—	73.6
Hatchlings (single embryo)	Mean	18	Female	121.2	56.8	8.20	129.40	—
		14	Male	117.3	55.3	8.10	125.40	—

*Note:* Embryos from twin and triplet eggs were connected to the same yolk and thus share the same yolk weight. Data available from Guzy et al. 2025.

**FIGURE 2:**
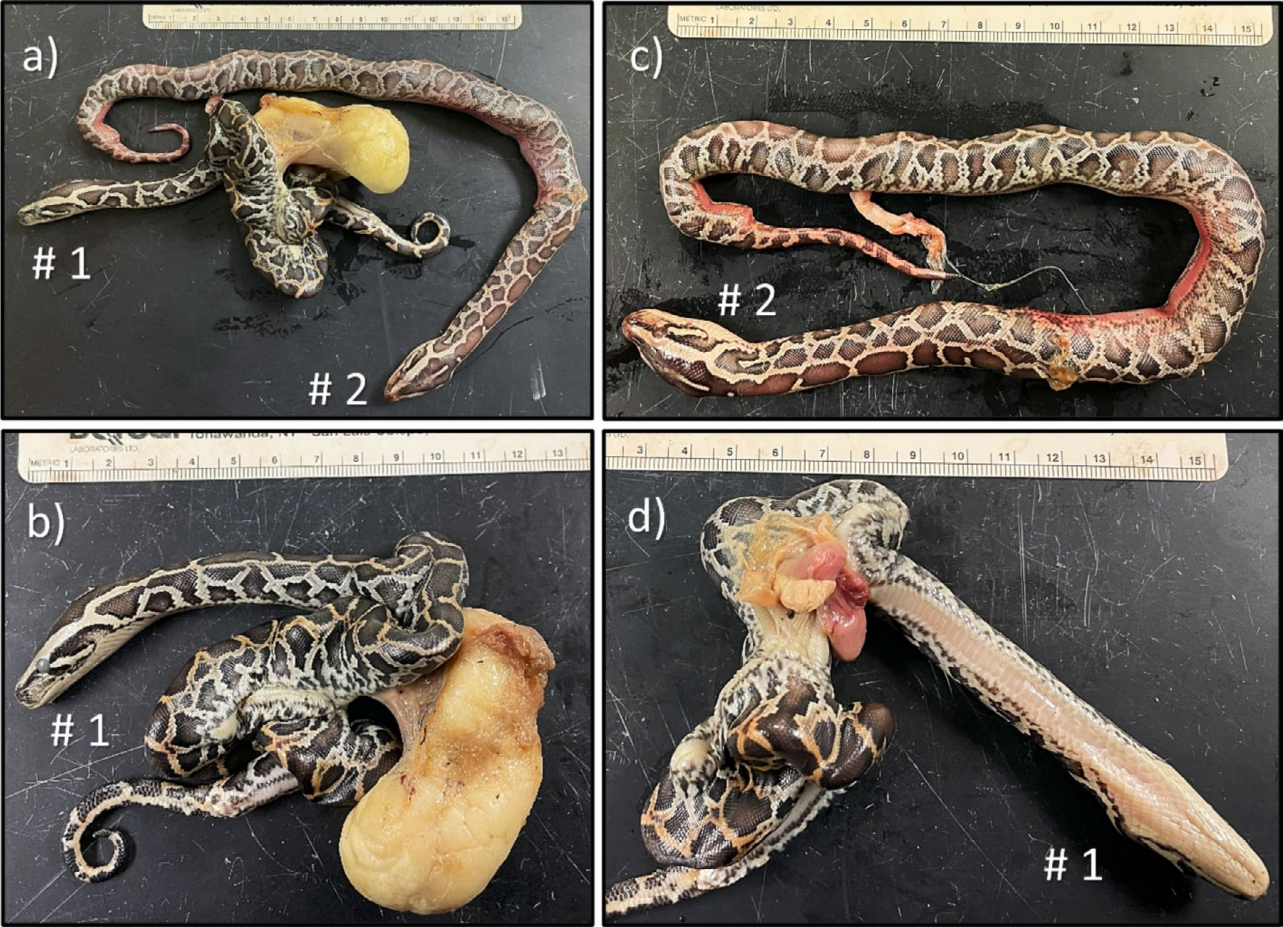
Twin embryos within one egg (Egg A) were oviposited by a wild adult female Burmese python in southern Florida. Eggs within this clutch began hatching on July 17th, 2024, and the clutch was continuously monitored until hatching was completed. This egg was observed on 22 July 2024, with a 2‐cm slit (i.e., hatching initiated). Both embryos were attached to the yolk and found deceased. Embryo #1 (a–c) was fully pigmented and had skeletal deformities, with conjoined organs growing outside of the lower third of the body (b, c). Embryo #2 (a, d) exhibited fully developed pattern and pigmentation and no organs outside the body.

**FIGURE 3:**
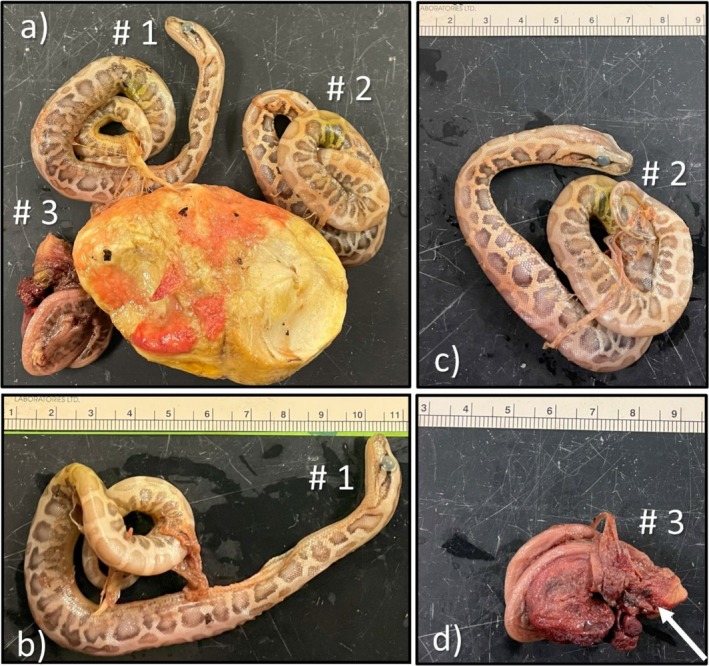
Triplet embryos within one egg (Egg B) oviposited by a wild adult female Burmese python in southern Florida. Eggs within this clutch began hatching on July 17th, 2024 and the clutch was continuously monitored until hatching was completed. All three embryos were attached to the yolk and found deceased. Embryo #1 (a, b) was underdeveloped with the beginning stages of pattern development and lacked pigmentation to skin. Embryo #2 (a, c) exhibited similar development as Embryo #1, both snakes exhibited abnormally large eyes but otherwise normal organs. Embryo #3 (d) had developed a slight pattern but was very underdeveloped, and the body was compressed within the egg, particularly around the head and neck regions (white arrow).

At the time of processing on 22 July 2024, Egg A exhibited a 2‐cm slit, indicating pipping had initiated, and inside, there were two well‐developed embryos (Figure 2; Twin #1 and Twin #2). The egg tooth on each snake was firm, indicating that at least one individual survived long enough to initiate pipping. Both snakes were female. Despite its later stage of development, Twin #1 had major skeletal and developmental issues (Figure 2a–c). At the center of the body, its spine was severely kinked, and the ventral sides were fused together. However, the anterior 7 cm of the body and the posterior portions 1.5 cm from the cloaca appeared to have developed normally. A necropsy revealed that the organs were compressed together, with the heart and a small portion of the lungs developing outside of the body cavity within the yolk. Despite this, Twin #1 was more pigmented than Twin #2, and both were similar in mass (Table 1), possibly indicating that they were developing at similar rates. A necropsy for Twin #2 indicated normal development with all organs intact (Figure 2a,d). Egg B was intact, and pipping had not initiated. Upon necropsy, three embryos were found in various stages of development (Figure 3). Although Triplet #1 and Triplet #2 were similar in size and weight, Triplet #3 was noticeably less developed, compressed within the egg, and weighed less than 6 g (Table 1). The sex of each triplet embryo was indeterminable; they were smaller than both twin embryos, and most of the yolk was unabsorbed (Figure 3). All embryos from Egg A and B were shorter and lighter in mass than their single‐embryo siblings (Table 1), likely because of resource sharing.

Twinning in reptiles can occur when a single fertilized ovum divides into two independent embryos (monozygotic twins), or when two fertilized ova, each with its own yolk sac, are enclosed within the same eggshell (dizygotic twins; Marion 1980; Dieckmann et al. 2014). Several morphological attributes have been proposed to distinguish between these forms of twinning. Dizygotic twins are hypothesized to exhibit size variation, and their eggs are believed to be larger than the rest from the same clutch because the singular egg must contain two separate yolk sacs (Rodríguez‐Cabrera et al. 2022; Dieckmann et al. 2014). In this study, Eggs A and B were similar in size to each other and to the other eggs in the clutch (Figure 1), and all embryos were connected to one yolk. Although it was not possible to compare the SVL lengths of all embryos within Eggs A and B because of severe deformities, the twins in Egg A had similar body masses (see Table 1). These observations suggest a possible monozygotic origin for Egg A. In contrast, one of the triplets from Egg B was much smaller than the rest, complicating the interpretation of its origin. These observations illustrate the limitations of relying on morphological traits alone to infer zygosity. Indeed, it has been hypothesized that yolk sacs may fuse to form a common yolk sac (Marion 1980), and there are no data thus far to demonstrate a strong link between size disparities and dizygotic twinning (Rodríguez‐Cabrera et al. 2022). Further genetic testing would be necessary to be definitive.

Burmese pythons exhibit multiple paternity, where more than one male can sire a clutch, either because of sperm storage by females across reproductive cycles or copulation with multiple sires during a single reproductive cycle (Skelton et al. 2021). Multiple paternity likely increases genotypic diversity via more combinations of alleles, but it is unknown if this may contribute to instances of twinning in this species. It is unclear how often multi‐embryo eggs occur in wild Burmese pythons; however, on the basis of our observations of wild pythons thus far, we suggest that it is uncommon. In May 2022, the female in this study oviposited a clutch of 19 viable and 1 inviable egg in the wild, which were subsequently removed or monitored as part of ongoing research, and none of these were multi‐embryo eggs. In the published studies of 13 wild Burmese python clutches in southern Florida, none have contained multi‐embryo eggs (Currylow et al. 2022); nor has this been seen in 12 additional clutches oviposited by wild females as part of ongoing research since 2021 (A. Yackel Adams and M. Sandfoss, U.S. Geological Survey, Written Communication, 15 May 2025). Here, we report the first instance of a single wild Burmese python laying eggs containing both twin and triplet embryos in wild Burmese pythons, and although none of these embryos survived to hatch, more research is needed to determine how prevalent this is in the wild population, the overall likelihood of survival to hatching, and resulting contributions to fecundity for this species.

## Author Contributions

**Génesis Aponte Santiago:** conceptualization (equal), data curation (equal), formal analysis (equal), investigation (equal), methodology (equal), writing – original draft (equal). **Judith E. Baird‐Lujano:** conceptualization (equal), data curation (equal), formal analysis (equal), investigation (equal), methodology (equal), writing – original draft (equal). **Jacquelyn C. Guzy:** conceptualization (equal), funding acquisition (equal), investigation (equal), project administration (equal), supervision (equal), visualization (equal), writing – original draft (equal). **Derrick G. Biglin:** investigation (equal), methodology (equal), writing – review and editing (equal). **John‐Kaarli M. Rentof:** investigation (equal), methodology (equal), writing – review and editing (equal). **Christina M. Romagosa:** funding acquisition (equal), investigation (equal), project administration (equal), resources (equal), supervision (equal), writing – review and editing (equal). **George F. Bancroft:** investigation (equal), methodology (equal), writing – review and editing (equal). **Matthew McCollister:** investigation (equal), project administration (equal), resources (equal), writing – review and editing (equal). **Kristen M. Hart:** funding acquisition (equal), investigation (equal), project administration (equal), supervision (equal), writing – review and editing (equal).

## Conflicts of Interest

The authors declare no conflicts of interest.

## Data Availability

All data associated with this manuscript are provided via Guzy et al. USGS Data Release. Citation: Guzy JC, Baird‐Lujano JE, Aponte‐Santiago G, and Hart KM. 2025. Length and mass measurements for a clutch of Burmese python hatchlings from southern Florida, including observations of multiembryo eggs. U.S. Geological Survey Data Release. Available at https://doi.org/10.5066/P13KRE2E.
